# The benefits and barriers to physical activity and lifestyle interventions for osteoarthritis affecting the adult knee

**DOI:** 10.1186/1749-799X-7-15

**Published:** 2012-03-31

**Authors:** Jonathan Daniel Stevenson, Richard Roach

**Affiliations:** 1 Department of Trauma & Orthopaedics, Princess Royal Hospital, Apley Castle, Telford, Shropshire TF1 6TF, UK; 2 6 Beaumont House, Old Stafford Road, Cross Green, Staffordshire WV10 7EP, UK

**Keywords:** Osteoarthritis, Knee, Exercise, Interventions, Barriers

## Abstract

Osteoarthritis prevalence is increasing, placing greater demands on healthcare and future socioeconomic costing models. Exercise and non-pharmacological methods should be employed to manage this common and disabling disease. Expectations at all stages of disease are increasing with a desire to remain active and independent. Three key areas have been reviewed; the evidence for physical activity, lifestyle changes and motivational techniques concerning knee osteoarthritis and the barriers to instituting such changes. Promotion of activity in primary care is discussed and evidence for compliance has been reviewed. This article reviews a subject that is integral to all professionals involved with osteoarthritis care.

## Introduction

Osteoarthritis (OA) is the most common form of arthritis, typically seen with increasing age affecting all joints. The majority of people over 60 years of age show evidence of osteoarthritis in at least one joint, with radiological evidence presenting in 70% of hips or knees of those older than 65 years [1]. The prevalence is 10% of 60 year olds and increases with age; the total number affected will have doubled by 2020. It is typified by morning pain, stiffness, swelling and deformity and risk factors include excessive force from repetitive impacts [2]. Body mass index is therefore also important. In addition, injury can also lead to abnormal strains upon individual joints [3]. Physical activity is limited by pain, eventually causing reduced muscle strength and atrophy. Features of Osteoarthritis section outlines typical features, which can be individually assessed and targeted for treatment.

## Features of osteoarthritis

History: Pain, swelling, stiffness, heat, limp, reduced activity.

Examination findings: Heat, pain, swelling, flexion deformity, weakness, restricted movement.

Radiographic changes: Osteophytes, subchondral sclerosis, subchondral cysts, joint space narrowing, deformity, soft tissue calcification, effusion.

Osteoarthritis has a significant socio-economic cost and therefore essential research is aimed at all levels of intervention and pathogenesis [4]. It is accepted that clinical function and radiological findings do not always parallel symptoms. For acute cases, management predominantly involves pain control, restoration of range of motion and swelling management. In the chronic state, a combination of pharmacological and non-pharmacological management exists. The latter includes muscle strengthening, range of movement therapy and aerobic conditioning in addition to occupational therapy, patella taping, weight loss, personalised social support, bracing, walking aids, and shock absorbent insoles [5].

Recent reviews of exercise with regard to osteoporosis, cardiovascular disease, diabetes, stroke, falls prevention and obesity have been published [6-13]. In 2008 the National Institute for Health and Clinical Excellence (NICE) published a framework of management concerning osteoarthritis [14]. It emphasised a holistic patient centred treatment, with local muscle strengthening and general aerobic fitness, without any further qualification or specificity to the knee. The Cochrane report from 2009 was similar but detailed a more specific regime for the osteoarthritic knee [15]. The authors concluded that the evidence to support exercise was convincing and that the magnitude of effect was comparable to non-steroidal anti-inflammatory medications.

## Prevention

Advice can be given on prevention to try and limit high loading to the knee in sporting activities e.g. rugby, football and weight lifting. Patients in occupations such as in particular mining and farming have also previously been identified as being at risk. Body mass index attributes to joint loading and should also be a focus of attention. Usually at an early stage there is no pain. Consequently, exercise and weight control advice becomes secondary prevention. This includes a variety of exercise techniques, examples are listed in Additional file 1: Figure S1.

### The evidence for physical activity

National Health Service (NHS) publications in 2009 'Let's get moving' and 'Be active, be healthy' both state that the British population is predominantly inactive [16,17]. This correlates strongly with disease, health risk, economic costs, loss of functional earning capacity and premature mortality. This epidemiological relationship forms the basis for health commissioning. Evidence for individual treatment of chronic osteoarthritis is sparse, frequently because it is difficult to control confounding variables or separate overlapping treatment modalities. Treatment is always individualised to the diseased joint and multi-factorial. Assessment tools are invariably weak and are not universal.

In an extensive review by Pederson and Saltin from 2006, strength training combined with endurance training was advocated for osteoarthritis [18]. Strengthening surrounding muscles enhances joint function and co-ordination, and endurance training helps with weight loss and general physical conditioning. The EULAR (European League Against Rheumatism) recommendations for knee and hip osteoarthritis separately outlined their own guidelines [19]. These focused on general, non-pharmacological, pharmacological, surgical and combination therapies. Quality of data was expert opinion based, evidence based, or an amalgamation of both. Authors reviewed 1,447 citations of which only 21 met their criteria. The summary closely agreed with the American College of Rheumatism (ARC) guidelines from 2003 but they decided to keep the knee separate from the hip due to differences in risk factors, anatomy, treatment options and use of topical agents. A review by Petrella in 2000 regarding effective exercise treatment for osteoarthritis reviewed 23 trials, of which only three were considered sufficiently powered to draw conclusions [20]. Petrella concluded that exercise programs should be offered to all patients as a mainstay of non-pharmacological management of knee osteoarthritis and that focusing on associated co-morbidities, in particular obesity, could also help with compliance. The typically responsive patient would present with mild to moderate osteoarthritis with limited function, pain and mild stiffness.

### The evidence for promoting lifestyle change

Published articles have evaluated how to promote lifestyle change with motivational interviewing techniques [21]. Jensen in 2003 suggested a self motivated pain management model [22]. Somers in 2008 described reducing abnormal pain behaviour (catastrophising) and responses to treatment for knee osteoarthritis [23].

In 2003 Estabrooks, Glasgow and Dzewaltowski provided recommendations for physicians in the role of physical activity (PA) promotion [24]. They surmised that the evidence was variable and not universally followed and they proposed the following principles. Firstly, intervention activity does not need to be time consuming nor senior-led. Secondly, patients need to be active participants in decision making, helping to develop these plans and strategies to overcome barriers and monitoring. Thirdly, feedback was critical and had to be specific. Finally, to ensure maintenance, intervention activity should be integrated into community opportunities wherever possible. The sequence of promotion in primary care was the five 'A's':

1. Assess the current level of physical activity, abilities, beliefs and knowledge

2. Advise on health risks, benefits of change, appropriate activity, its quantity and intensity

3. Agree a personal developmental plan with appropriate goals

4. Assist in identifying barriers and strategies to address these. Also to link in with community opportunities for activity and social support

5. Arrange follow-ups by telephone calls or letter.

A cross sectional study published in 2009 by Scopaz *et al. *clarified psychological factors and their influence upon physical function with 182 patients with knee osteoarthritis [25]. They looked at anxiety, depression and 'pain fear' avoidance using a range of functional and psychological scores. Critically they used only one activity of daily life score and one generic lower limb joint pain and function score. Other tools exist which are more specific to the knee, which could have been used to improve validity and comparison.

Scientific literature from the last ten years identified seven randomised control trials (RCTs) concentrating on activity interventions for knee osteoarthritis. These have strengthened the case for activity intervention concentrating on all aspects for example hands-on treatment [26], primary care exercise advice [27], health education [28] and group versus home exercise [29]. These studies also gave the impression of better results with longer intervention periods of attendance but the maximum study follow up was 18 months. Comparing all the RCTs relating to exercise and lifestyle interventions in knee osteoarthritis, significant variance existed in sample sizes, length of follow-up, assessment tools with often minimal description in disease severity.

In the 'Arthritis, Diet and Activity Promotion Trial', 206 patients were randomised into diet and exercise interventions over 18 months [30]. Early compliance to dietary intervention occurred in isolated cases, following one advice session. Longer compliance with advice resulted from home exercise plans with single advice sessions. The authors concluded that compliance was related to a location of exercise and an emphasis on a stimulating early intervention session attendance.

The ARTIST (Osteoarthritis Intervention Standardised Trial) study concentrated on the impact of consultations by primary care rheumatologists in France [31]. These patients had either three standardised consultations or normal treatment. At four months, both weight loss and physical exercise levels were significantly improved in the consultation group. At one year, function and pain levels were also improved. This study was well executed as it excluded significant co-morbidities and ensured osteoarthritis was the definitive diagnosis. The interventions were one-to-one and concentrated on self confidence, barriers, and methods of overcoming these and goal setting. The trial designers accepted that time was limited and, as such, communication had to be effective. Each of the three visits was therefore carefully controlled; the first visit concentrating on the disease and treatment with the second and third on exercise or weight loss in either order. The interview was motivational but not, however, clearly defined in the study. Advice regarding weight control did follow government published guidelines: the patient had to assess their own risk and be ready to lose weight and determine a programme and appropriate strategy. Numerous booklets were also provided to patients. The outcomes were independently determined. The study also formally studied appreciation and consequences to intervention as judged by knowledge retention which was improved in all cases, but clearly short-term. Limitations included the blinding of the study, the wish for matched groups but the control group had heavier patients and nine cases were excluded. Interestingly, the mean weight loss was only one kilogram at 4 months with no difference between groups at one year.

The social cost has also been studied by Sevick *et al. *in Pittsburgh [32]. This was a single-blinded, controlled trial of 316 adults with knee OA, randomised to one of four groups: Healthy Lifestyle Control group, Diet group, Exercise group, or Exercise and Diet group. Combined exercise and dietary intervention demonstrated the greatest benefits in weight control, symptom control and physical function levels and also proved to be the most cost-effective strategy.

Three reviews have been published specific to influences of exercise on osteoarthritis of the knee [33-35]. They found that the short-term benefits of aerobic and strength training for pain and function are not borne out in the long term (greater than 6 months). They reiterated the comments above of the lack of clarity with a Cochrane review on musculoskeletal pain recommending standardised assessment tools and longer follow-ups to identify long-term strategies for physical activity in knee osteoarthritis.

## Barriers to activity participation

Additional file 1: Figure S2 lists typical facilitators and barriers to activity participation [36-38]. This is important as it helps us to understand social behaviour change. It also gives clues as to how to bring about change, in what setting, and how to maintain it. It is accepted that this is multi-factorial based on numerous factors for example psychological status, environment, gender, age, condition and the influence of primary health care professionals [39-42].

Parfitt and Gledhill emphasised that choice is linked to self-determination and hence the individuals rating of perceived exertion (RPE) [43]. Consequently, this affects the intensity level and the ultimate psychological effect. Previous studies affirm that high intensity alone caused a change in effect but it is now suggested that there is more of a dose response phenomenon. There are quoted 'Exercise Benefits/Barriers scales' as assessment tools. This is outlined in EBBS: The exercise benefits/barriers scale section [44]. Response can be graded 1 to 4 using a Likert format (1 strongly disagree, 2 disagree, 3 agree, 4 strongly agree).

### EBBS: The exercise benefits/barriers scale

Exercise Milieu subscale: Location too far away, Embarrassment, Cost, Convenient, Appearance, Too few places to exercise.

Time expenditure: Takes too much time from relationships, Takes too much time from responsibilities, Takes too much of own time.

Exertion: Tiring, Gets fatigued, Is hard work.

Family discouragement: Spouse does not encourage, Other family members do not encourage.

Counselling can take place in a variety of places such as General Practice (GP), age care facilities, the work place, schools, university or as part of an occupational therapy return to work programme. The latter has worked very well as part of the Dutch 'Gatekeeper Act' 2002 whereby the employer and rehabilitation service have an equal responsibility to facilitate recovery under the control of an occupational therapy physician. This lasts one year and if the employer defaults or the individual does not comply then penalties ensue. From the socio-economic perspective this has, overall, been met with a good response.

The most recent reference concentrating on General Practitioners, encouraging health and exercise in older adults in Manchester, confirmed there was little difference between South Asian and white communities [45]. They underlined that guidance should be tailored to individuals, prescription exercise has been met with a good response, and that GP led advice could initiate activity in 60-70 year olds. The authors concluded that General Practitioners were best placed to offer lifestyle recommendations. This would create a dramatic cultural change as part of the Government's drive for a fit nation. General Practitioners would require support to maintain compliance with advice, perhaps in the form of information booklets with specialist nurse or physiotherapist led consultations. General Practitioners may not be aware of the evidence nor have sufficient time to deal with 'well' individuals.

## Facilitating lifestyle behaviour change for physical activity

The Transtheoretical Model of Behaviour Change (TTM) was originally used in the 1980s to describe behaviour change in the context of smoking cessation [46]. Critical to participation in activities associated with addiction is motivational interviewing during the consultation, according to Miller [47,48]. It was understood that behaviour was a process rather than a single event, and as it involved a number of parameters, was seen as being 'multifactorial', hence transtheoretical. The proposed model involved a series of stages outlined in Additional file 1: Figure S3 [49].

It was accepted that individuals could present at any level and maintain this level for a variable period of time. The principal difficulties relate to contemplating initiation of sporting or other activities. Unfortunately 50% of cases that do initiate activity are unable to maintain this beyond six months; therefore psychology is important to maintain activity. Additional file 1: Figure S4 and the positive factors for building confidence section below outline factors used by patients to support or refute the argument of adopting physical activity and confidence, which also contribute to maintenance [50-56].

### Positive factors for building confidence

Previous success, Memory, Role models, Trainers, External support, Arousal level, Genetics, Repetition, Self belief, Motivation, Financial incentives, Recognition, Career plans, Self satisfaction.

Relapses should be anticipated and be dealt with optimistically by evaluating the reasons and building a new strategy. The aims of consultations are therefore to motivate, plan and prevent relapse, as summarised by Schoo [57]. Empowerment involves allowing the individual to unlock his/her motivation and determine barriers. Reducing ambivalence or contradictions must be identified and dealt with. Any resistance should be combated with evidence and direction for the patient to resolve them. This is helped by transferring information. Regular summaries help clarify understanding and key points during the consultation. The consultant should also occasionally summarise opinions and support conflicts with evidence. After exploring all aspects the interviewer should summarise details as a 'Turning point' to determine an outcome: 'so what shall we do then.' If this is not possible during the consultation, this should be accepted temporarily, referred to as 'Letting go'. The seeds of thought, however, have been laid and a further appointment can then be made. Planning or goal setting and contracts may also be agreed. The final summary is 'the close'. A follow-up meeting should be arranged to deal with all outcomes both negative and positive.

The consultation can be assessed as outlined by the BECCI (Behaviour Change Counselling Index) [58]. The principles are that it should be patient centred, open-ended, supportive, non-direct and reflective.

## Planning/Goal setting

Goal setting has been applied to all aspects of life: industry, sciences, sports, self control and management. Industry aims for a target outcome whereas sports aims for maximal performance, which is ideally the maximum potential, Steps in goal setting include awareness of the pre-injury status, a list of goals that a gymnast would be likely to achieve and time scales, analysis of the current state, selection of appropriate goals at each time frame and a formulation of results and feedback. Teamwork can still be applied to individual sports as it depends on non-competing persons such as family, coach, psychologist and school.

Types of goal can include 'outcome', 'performance' and 'process' goals, for example the athlete's particular techniques used [59,60]. The emphasis is predominantly performance, repetitive practice, positive thought processes and participation to build confidence. This is especially the case following injury [61,62]. Goals can be at multiple levels and both short and long-term. The type of sport dictates the emphasis of these. In team sports, individuals may focus on the scoreboard whereas they should be concentrating on 'performance' aspects. This is can also be relevant to the recovering gymnast; working on safe routines to score well, rather than risky 'more to lose' techniques. Goal settling principles have been described using the acronym SMARTER, as outlined in Table 1[63]. Psychologists suggest that multiple goals at variable levels as a continuum are better than targeting just one particular standard.

**Table 1:** The SMARTER regime of goal setting

S	Specific	A target. No uncertainty
M	Measurable	Quantifiable
A	Achievable	Reasonable goal that prompts compliance
R	Realistic	Real and appropriate
T	Time contingent	Correct timeframe
E	Exciting	Stimulating
R	Re-evaluated	Should evolve with time

### Appreciation and consequences in patient understanding

Few studies have been specific to osteoarthritis of the knee and concentrated on the process of interventional techniques and patient understanding. The ARTIST study did make a specific trial intention to clarify this further but follow-up was brief with patients recalling co-morbidity, weight loss and activity advice early on, but only general osteoarthritis advice at one year [64]. A recent review by Breckon, Johnson and Hutchison from 2008 examined physical activity counselling content and competency and concluded this is a common fault with trials [65]. Out of 27 studies few covered this area clearly and none make reference to knee osteoarthritis. This is surprising considering the more widespread policy of white papers regarding activity intervention.

## Conclusions

Despite International and Government initiatives, there is little time, limited budgets, poor understanding, variable standards and no specific training to disseminate knowledge [66]. Breckon refers to this situation as studies stating 'what but not how', 'theory and not practise' and 'outcome not process'. Motivational interviewing is well founded in disease behaviour modification, as stated by Rubak *et al. *in a meta-analysis from 2005 [67]. However studies concerning other chronic diseases such as the American Heart Foundation statement from 2006, state that Randomised Control Trials remain poor to date [68].

Which discipline could take up the challenge of adult osteoarthritis of the knee? Public health officer, Doctor, Nurse, Sports diploma doctor, physiotherapist, personal trainer or a newly trained counsellor? It should be multidisciplinary, but not 'together under one roof'. A counsellor is a good idea, but senior level advice may carry more authority and response. If the whole chain of management used standardised pathways it would work well within existing frameworks. Knee osteoarthritis could be managed by a General Practitioner in a way similar to the French model with nurse led weight reviews, orthopaedic surgeon screening reviews, non-operative care, lifestyle physiotherapists or community therapy sessions maintaining regular exercise. Anecdotally, local social gym-based initiatives offered via primary are usually well received if at suitable times. Many local authorities now offer free swimming to over 45 year-olds, again at certain times in the day. Flexibility is required as patients present with different severities of disease, levels of expectation, compliance, receptiveness and timescales. The practice of partnerships in care and pathways could deliver the most practical way of managing this, as suggested by the recent commentary by Lianov and Johnson [69]. Research must be standardised, longer-term and more robust to ascertain the way ahead.

## Abbreviations

OA, Osteoarthritis; NICE, National Institute of Health and Clinical Excellence; EULAR, European League Against Rheumatism; ARC, Arthritis Research Council; PA, Physical Activity; RCT, Randomised Control Trial; ARTIST, Osteoarthritis Intervention Standardised Trial; RPE, Rating of perceived exertion; GP, General Practice; TTM, Transtheoretical Model of Behaviour Change; BECCI, Behaviour Change Counselling Index.

## Competing interests

All named authors hereby declare that they have no competing interests to disclose. This review received no specific grant from any funding agency in the public, commercial, or not-for-profit sectors.

## Authors' contributions

JDS wrote the manuscript and assisted in editing. RR performed the literature research and editing. Both authors read and approved the final manuscript.

## Supplementary Material

Additional file 1 **Types of exercise recommended: a variety of terminology**. Facilitators and barriers to change [36-38]. Stages of the Transtheoretical Model of Behaviour Change. Factors used to support or reject sport participation [50-54]. Click here for file
