# Supplementary material for: The benefits and barriers to physical activity and lifestyle interventions for osteoarthritis affecting the adult knee
Source: J Orthop Surg Res. 2012 Mar 31;7:15. doi: 10.1186/1749-799X-7-15 (PMC3353175; doi:10.1186/1749-799X-7-15)
Supplement: Additional file 1 — Types of exercise recommended: a variety of terminology. Facilitators and barriers to change [36-38]. Stages of the Transtheoretical Model of Behaviour Change. Factors used to support or reject sport participation [50-54]. [file 1749-799X-7-15-S1.DOC]

**Figure S1 Types of exercise recommended: a variety of terminology**

| **Resistance work** | **Low-impact** |
| --- | --- |
| Aerobic | No-impact |
| Yoga | Gravity assisted |
| Pilates | Isokinetic |
| Tai Chi | Light weight training |
| Group exercise/ home based | Walking |
| Balance control | Brisk walking |
| Sitting/ standing exercise | Hydrotherapy |
| Range of motion | Thermotherapy |
| Shock absorbent insoles | TENS |

**Figure S2 Facilitators and barriers to change [**[**36**](#CR36)**-**[**38**](#CR38)**]**

| **Facilitators** | **Barriers** |
| --- | --- |
| Belief that will improve life | Fatalism |
| Relevance | Denial |
| Good self efficacy | Poor efficacy |
| Low intensity | No history of exercise |
| Suggested by health professional | Fear of injury |
| Involved in destiny | Poor health |
| Social support | Low health expectations |
| Educated | Stigma |

**Figure S3: Stages of the Transtheoretical Model of Behaviour Change**

Action

Preparation

Maintenance

Relapse

Contemplation

Maintenance

Pre-contemplation

Stable Behaviour

**Stages of the Transtheoretical Model of Behaviour Change**

- Pre-contemplation: inactive. No intention to change
- Contemplation: inactive: intention to change in next 6 months
- Preparation: engaging but not regularly
- Action: regular but less than 6 months
- Maintenance: more than 6 months
- Termination

**Figure S4 Factors used to support or reject sport participation [**[**50**](#CR50)**-**[**54**](#CR54)**]**

| Positive | Negative |
| --- | --- |
| Enjoyable | Cost |
| Non-inhibitory | Time |
| Confidence | Weather |
| Success | Boredom |
| Positive self feeling | Lifestyle |
| Social support | Lack of energy |
| Perceived benefits | Medical state |
|  | Injury |
|  | Perceived low fitness level |
|  | Choice of sport |
